# Pharmaceutical pricing and reimbursement policies: perspectives for the future

**DOI:** 10.1186/2052-3211-8-S1-K3

**Published:** 2015-10-05

**Authors:** Andrew L Gray

**Affiliations:** 1 Division of Pharmacology, Discipline of Pharmaceutical Sciences, University of KwaZulu-Natal, Durban, 4041, South Africa

## Setting the scene

For many years, the global approach to pharmaceutical pricing and reimbursement policies has been informed, first and foremost, by the key differences between those systems which relied on public sector procurement and supply, and those which were based on reimbursement.

The first approach was considered to be the most appropriate for developing countries. Funding for healthcare services was based on disbursement from the fiscus, often exceeded by funds obtained from donors or development partners, and supplemented by considerable out-of-pocket payments in the form of user fees. In such systems, where medicines were provided by the state, these were often procured by a central medical store, and then distributed to public sector clinics and hospitals. Pricing interventions were limited to the application of limited competitive bidding, for a list of medicines determined centrally and severely limited. In some, but not all cases, this national essential medicines list was informed by the WHO Model List of Essential Medicines, updated approximately every 2 years since 1977. While procurement of generic medicines was the norm, a sophisticated generic substitution policy was often not in place. Generic prescribing was preferred, but rarely practised. Rational use of medicines was expected to follow, almost automatically, but rarely pursued with much vigour [1]. For many of these countries, stimulating a local manufacturing industry has either been irrelevant or of subsidiary interest. Locally relevant innovation has been reliant on external funding, largely delinked from local pharmaceutical policies.

The second approach, applied most vigorously in those countries with national or social health insurances system, was appropriate to systems in which the financing of health care was separated from the provision of services. In relation to medicines in particular, a wider range of policy options were implemented, including a variety of measures to promote generic medicines use, the use of co-payments and other risk-sharing options, external reference pricing, distribution chain price controls and health technology assessment. In many such countries, health policy has had to co-exist, if not seamlessly dovetail with industrial policies aimed at protecting local manufacturing. Innovation has been almost exclusively driven, at least after initial public support, on the protection and exploitation of intellectual property. However, the applicability of many of these pharmaceutical policies to low- and middle-income countries has been questioned [2].

The challenge for the future is to identify a range of pharmaceutical pricing and reimbursement policies that are both appropriate for and supportive of countries' attempts to introduce and entrench universal health coverage. They will also need to stimulate necessary and appropriate innovation, while ensuring a responsible and stable pharmaceutical industry, in alignment with national and regional industrial policies. This is a tough call, which calls for a delicate balancing of many disparate interests, in a way which is also patient-centred and cognisant of the human rights at stake.

## South Africa – an exemplar

South Africa has been engaged in the implementation of a National Drug Policy since 1996, with highly publicised challenges mounted by the pharmaceutical industry to the initial interventions [3]. Those early interventions included the introduction of mandatory offer of generic substitution, a ban on all samples of medicines, a non-discriminatory single exit price (factor gate price) for medicines and a ban on volume discounts, a maximum annual percentage increase in the single exit price, and a maximum dispensing fee for pharmacists and other dispensing practitioners. The policy has aimed to achieve ‘transparency’ in the pricing of medicines, but has failed to achieve this in relation to the logistics fee paid to wholesalers and distributors by manufacturers, and included in the single exit price. Although nationally-representative data are elusive, it appears that the generic share by volume (of prescriptions) is now in the mid-50 % range, and rising very slowly. However, there are concerns that systematic attempts have been made to circumvent the ban on volume discounts, and to create incentives for large buyers in the form of data fees, co-marketing fees and other forms of off-invoice bonuses. International benchmarking (reference pricing) has been repeatedly signalled, but as yet not implemented. The submission of pharmacoeconomic data to justify launch prices has been introduced, but remains voluntary. New, expensive, and often biological, products are placing an increasing burden on the medical schemes that serve the insured population.

Critically, the pricing and reimbursement policies listed above apply only to the private sector, which caters for barely 17% of the population (excluding those who pay out of pocket) [4]. The public sector still relies on local competitive tenders, predominantly with a single supplier per product delivered to public sector health facilities either via central provincial stores or directly. These products are identified by means of a national Essential Medicines List, based on comprehensive standard treatment guidelines. The public sector-dependent population is not able to access medicines via the private pharmaceutical infrastructure of community pharmacies and private hospitals, but has to rely on over-stretched and poorly resourced public sector facilities. The Minister of Health issued a Green Paper on National Health Insurance in 2011 [5]. A final policy document has not yet been issued, but an indication has been given that the implementation process will take up to 14 years. Among the challenges facing South Africa's attempt to introduce universal health coverage will be the need to move from a public sector selection, procurement and supply system to one based on reimbursement and a typical insurance-style purchaser-provider split.

At the same time, South Africa is home to the largest pharmaceutical manufacturing capacity on the African continent. Local preference procurement policies underpin the local industrial policy. Efforts to create an active pharmaceutical ingredient manufacturing capacity are underway. South Africa is also trying to reform its intellectual property system to be more critical and appropriate [6].

## Perspectives for the future

If recent product launches and the prices demanded are to be taken as a signal, and combined with the trend towards individualised medicine, then health systems in all countries are facing an insupportable demand for additional resources.

There is much interest in expanding the process of health technology assessment to low- and middle-income countries. In part this may be achieved through greater transparency, data sharing and the publication of models that can be repopulated with locally-determined cost data. However, what will be critical is the application of this suite of methods to the selection and appropriate pricing of the bulk of reimbursed medicines, as well as to new and expensive medicines.

Much more attention will need to be paid to the responsible use of medicines, and to systems which allow for a reliable estimate of the value of medicines under typical use. Whether that will enable widespread use of performance-based pricing remains to be seen. What is critical is that performance-based pricing must not provide a fig leaf behind which unacceptable launch prices can be hidden. Reimbursement policies and processes will also need to measured against their effects on responsible use, and adjusted where their effects are shown to be perverse and not in the interests of patients. Lastly, as was signalled strongly at the onset of this conference, consideration will need to be paid to the effect of pricing and reimbursement policies on necessary and appropriate innovation. Standing still is not an option, and complacency is entirely unwarranted.
